# Healthy n-6/n-3 fatty acid composition from five European game meat species remains after cooking

**DOI:** 10.1186/s13104-015-1254-1

**Published:** 2015-06-27

**Authors:** Teresa G Valencak, Lisa Gamsjäger, Sarah Ohrnberger, Nicole J Culbert, Thomas Ruf

**Affiliations:** Department of Integrative Biology and Evolution, Research Institute of Wildlife Ecology, University of Veterinary Medicine, Vienna, Savoyenstrasse 1, 1160 Vienna, Austria

**Keywords:** Game meat, Fatty acid composition, Kitchen processing, n-3 Fatty acids, n-6 Fatty acids, n-3/n-6 Ratio

## Abstract

**Background:**

Intensive farming of livestock along with recent food scandals and consumer deception have increased awareness about risks for human nutrition. In parallel, the demand for meat obtained under more natural conditions from animals that can freely forage has largely increased. Interestingly, the consumption of game meat has not become more common despite its excellent quality and content of polyunsaturated fatty acids (PUFAs).

**Context and purpose:**

We addressed the question if game meat fatty acid composition is modified through kitchen preparation. By analysing muscle fatty acid (FA) composition (polar and total lipids) of five European game species in a raw and a processed state, we aimed to quantify the proportion of PUFA that are oxidised and hydrogenated during processing. All game meat species originated from local hunters and free-living individuals. To mimic a realistic situation a professional chef prepared the meat samples with gentle use of heat in a standardised way.

**Results:**

Expectedly, the overall content of polyunsaturated fatty acids declined during the cooking process but the decrease size was <5% and the nutritiously most important n-3/n-6 ratio was not affected by processing (F_1,54_ = 0.46; p = 0.5). Generally, our samples contained species-specific high PUFA and n-3 FA contents but we point out that differentiating between species is necessary.

**Conclusion:**

Game meat thus provides a healthy meat source, as cooking does not substantially alter its favourable fatty acid composition. Further research is needed to elucidate species-specific differences and the role of habitat quality and locomotion for tissue composition.

## Background

Polyunsaturated fatty acids (PUFAs) are essential dietary components, because humans cannot synthesize them de novo. Dietary PUFA, in particular long chained n-3 fatty acids (FA), such as docosahexaenoic acid (DHA) and eicosapentaenoic acid (EPA) are well known for their beneficial health effects [1–3]. A shift in diet composition, especially over the last 50 years [see 4], has occurred and resulted in a higher intake of fat, specifically saturated fatty acids (SFA). Dietary fat levels have been heavily linked to lifestyle diseases, in particular coronary heart disease [5], resulting in the development of specific dietary guidelines [6–8]. The German Society for Nutrition (DGE) [8] recommends that fat should make up no more than 25–30% of an individual’s daily energy intake with SFA limited to below 10%. The n-6/n-3 ratio is commonly used as an index to evaluate the nutritional value of dietary fat that has particular relevance on human health. Today, Western diets are rich in SFA and n-6 PUFA and relatively low in n-3 PUFA. Increased n-3 consumption has been shown to have protective effects on a whole host of diverse conditions from arteriosclerosis to inflammatory and autoimmune diseases [9]. The Department of Health of the United Kingdom [6] set a recommended ratio of PUFA:SFA (P:S) at no less than 0.1 coupled with a n-6/n-3 ratio of below 4. Our predecessors, in ancient times, likely took up a n-6/n-3 PUFA ratio of around 1:1, based on a diet composed mainly of wild animal meat and plant matter [10]. This is a far cry from a modern Western diet, which ranges from an unfavourably high level of 10–20:1 [11]. By including more n-3 PUFA in our diets, we could actively reduce this ratio which would in turn, greatly improve our health.

It has been argued that plummeting levels of dietary n-3 PUFA are mainly attributable to a lack of fatty fish [12]. As it stands, fatty marine fish represent one of the most important sources of dietary n-3 PUFA in the Western world [13]. However, fish consumption in western societies is well below recommended levels [14, 15] and is fraught with potential problems, including the risk of an elevated intake of heavy metals, polychlorinated biphenyls, or dioxin [2, 16] through bioaccumulation. Additionally, an ever-growing demand for fish and fish oil is responsible for severe overfishing with dwindling fish stocks worldwide [17], coupled with various other detrimental effects on the marine ecosystem [18]. A booming aquaculture industry dominates the fish oil market as a prerequisite component of farmed fish food [19] whilst an ever-growing human population maintains the demand for n-3 PUFA from fatty fish. It is therefore of high importance to seek sustainable dietary sources of n-3 PUFA to meet and maintain our recommended intake and perhaps shift the n-6/n-3 PUFA ratio closer towards the 1:1 level as likely seen in our ancestors. Marine algae as well as engineered transgenic plants are being tapped to offer a sustainable source of n-3 PUFA in the coming years [20, 21].

Current trends show that consumers are becoming increasingly interested in meat quality and safety. Health conscious consumers have driven the demand for low fat and low cholesterol meat upwards in turn sparking a new interest in suitable alternatives to traditionally sourced farm meat products. One such alternative source is game meat [22]. Game meat in general has a low lipid content, typically less than 3%, in comparison to meat from livestock. In addition, lipids in muscles from game animals are dominated by structural lipids (i.e., phospholipids and cholesterol) with very desirable FA profiles and a low percentage of intramuscular fat (IMF) [10, 22, 23]. Previous analyses have failed however, to address the impact of processing on the FA content and composition of game meat: nonetheless processing is known to reduce the PUFA content in beef due to oxidation and hydrogenation of FA [24]. We hereby refer to the term processing throughout this contribution as the way of readying meat for human consumption, by means of cooking. For our present contribution, a professional chef from a restaurant in Austria that is specialised on game meat carried out the processing with the smallest possible addition of oil of the game meat and with the lowest possible variation according to his in-depth training and knowledge. Additionally, the restaurant obtains the game meat from the same, local hunters year round. We aimed to investigate whether game meat, even after processing, represents a valuable nutrient for human consumption, particularly with respect to its content of PUFA and the n-6/n-3 FA ratio. By analysing both muscle phospholipid (PL) and total lipid (TL) FA composition of samples from five European game species in a raw and a processed state, we attempted to quantify the proportion of FA oxidised and hydrogenated during processing.

## Results and discussion

**Figure 1:**
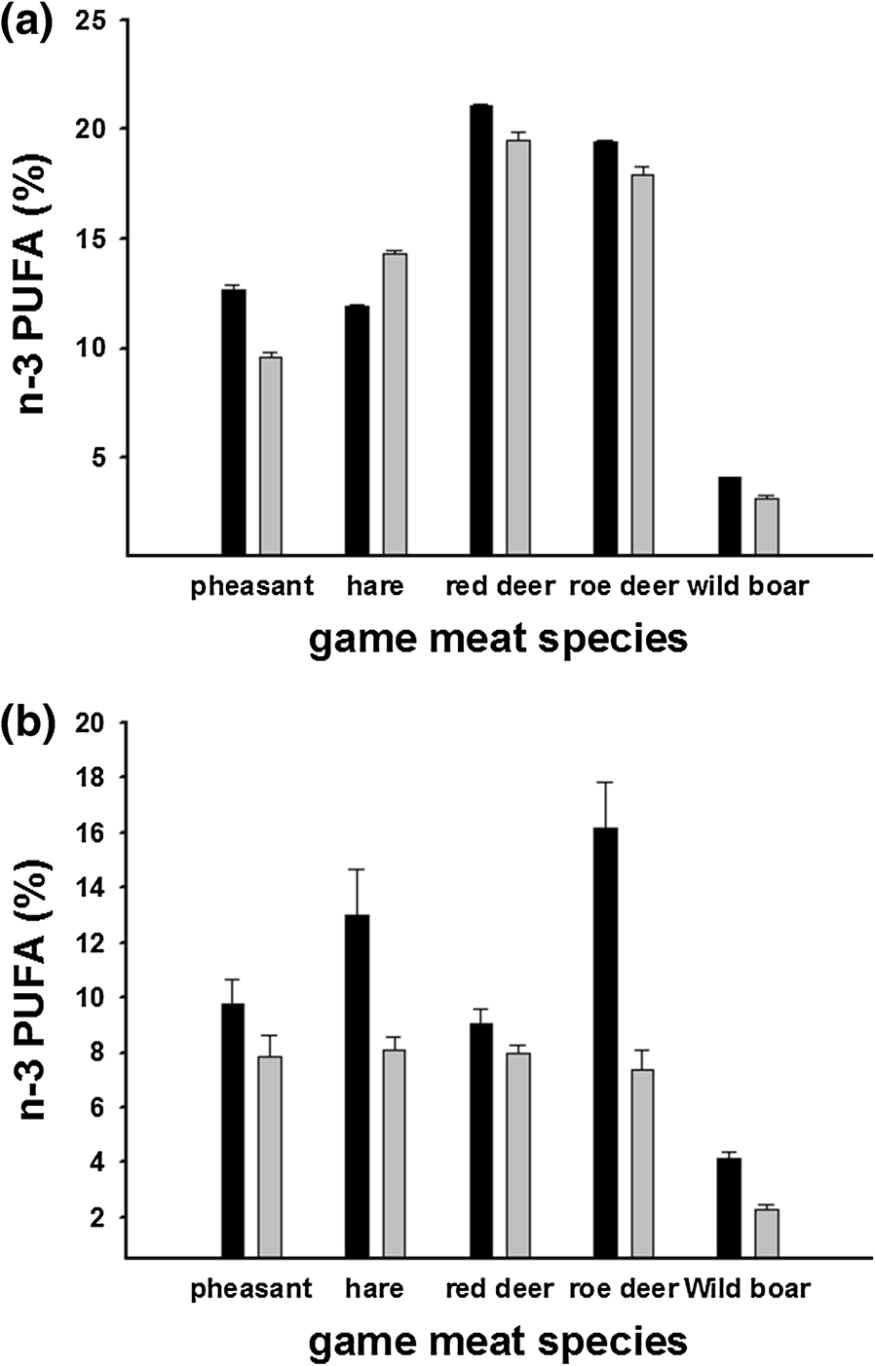
Phospholipid (**a**) and total lipid (**b**) n-3 PUFA (%) content of five common game meat species before (*black bar*) and after (*grey bar*) cooking.

Meat PL from hare, red and roe deer contained more than 60% total PUFA (and >55% in the processed state), while samples from wild boar and pheasant showed somewhat lower PUFA content (Table 1). Proportions of n-3 PUFA were high (ca. 60%) in meat from red deer, roe deer and hare (Figure 1a), but lower (40–50%) in pheasant and wild boar (Figure 1a; Table 1). FA composition was thus significantly different between the species studied (PUFA: F_4,54_ = 651.9; p < 0.0001, n-3: F_4,54_ = 447.7; p < 0.0001). Interestingly, variation between individuals, as can be deduced from low standard errors of the mean, was very low in all species (Table 1; Figure 1a, b). In all five species, the proportion of SFA was similar, but monounsaturated FA (MUFA) varied considerably, with increased levels in species with low PUFA content (e.g. pheasant and wild boar, Table 1). As expected, processed game meat had less total PUFA in comparison to raw samples (F_1,54_ = 179.01; p < 0.0001), albeit to a minor extent. Importantly, the cooking process had no significant effect on the n-6/n-3 ratio (F_1,54_ = 0.46; p = 0.5). Similar to PL, FA composition of TL was also significantly different between the species studied (PUFA: F_4,46_ = 7.48; p < 0.0001, n-3: F_4,46_ = 17.989; p < 0.0001, Table 2). Again, the cooking process decreased the amount of PUFAs (F_4,46_ = 45.672; p < 0.0001).

**Table 1:** FA composition of PLs extracted from pheasant, hare, red deer, roe deer and wild boar in a raw (R) and a processed (P) state; values are given in weight% with means ± SEM

	Pheasant		Hare		Red deer		Roe deer		Wild boar	
	R	P	R	P	R	P	R	P	R	P
N	6	6	6	6	6	6	6	6	6	6
C 14:0	0.3 ± 0.009	0.22 ± 0.02	0.4 ± 0.04	0.3 ± 0.04	0.7 ± 0.05	0.6 ± 0.07	0.3 ± 0.03	0.2 ± 0.007	0.2 ± 0.02	0.4 ± 0.02
C 15:0	0.03 ± 0.002	0.03 ± 0.004	0.2 ± 0.003	0.13 ± 0.005	0.3 ± 0.003	0.3 ± 0.005	0.3 ± 0.05	0.3 ± 0.004	0.1 ± 0.002	0.1 ± 0.09
C 16:0	20.2 ± 0.6	22.1 ± 0.3	14.4 ± 0.07	15.4 ± 0.2	12.5 ± 0.09	13.7 ± 0.3	9.5 ± 0.2	11.6 ± 0.08	18.6 ± 0.4	19.6 ± 0.9
C 17:0	0.08 ± 0.002	0.09 ± 0.002	0.8 ± 0.004	0.5 ± 0.002	0.8 ± 0.006	0.8 ± 0.009	0.6 ± 0.004	0.6 ± 0.003	0.6 ± 0.01	0.6 ± 0.01
C 18:0	16.8 ± 0.2	17.2 ± 0.3	15.9 ± 0.09	15.7 ± 0.2	16.5 ± 0.1	17.01 ± 0.2	17.9 ± 0.1	18.4 ± 0.1	13.6 ± 0.1	16.1 ± 0.8
C 16:1n-7	2.2 ± 0.3	1.4 ± 0.03	0.3 ± 0.005	0.4 ± 0.03	1.7 ± 0.04	1.8 ± 0.03	0.9 ± 0.07	0.8 ± 0.01	0.5 ± 0.1	1.3 ± 0.1
C 18:1n-9	19.5 ± 1.0	21.9 ± 1.1	5.9 ± 0.1	11.8 ± 0.6	6.7 ± 0.1	8.2 ± 0.3	4.3 ± 0.03	6.1 ± 0.7	7.9 ± 0.09	8.2 ± 0.2
C 18:2n-6	17.6 ± 0.6	18.6 ± 0.1	33.5 ± 0.4	28.02 ± 0.4	29.2 ± 0.3	28.7 ± 0.5	27.4 ± 0.3	27.7 ± 0.2	42.8 ± 0.2	40.2 ± 0.7
C 18:3n-3	0.3 ± 0.02	0.8 ± 0.08	3.4 ± 0.01	5.2 ± 0.09	10.4 ± 01.	10.2 ± 0.1	4.9 ± 0.09	5.3 ± 0.07	0.8 ± 0.02	0.7 ± 0.06
C 20:4n-6	10.7 ± 0.1	8.97 ± 0.2	16.9 ± 0.2	13.5 ± 0.2	10.5 ± 0.3	9.3 ± 0.5	19.5 ± 0.2	16.4 ± 0.4	11.5 ± 0.08	10.4 ± 0.8
C 20:5n-3	0.9 ± 0.02	0.7 ± 0.01	2.4 ± 0.06	2.3 ± 0.07	4.4 ± 0.07	3.9 ± 0.2	6.4 ± 0.07	5.4 ± 0.2	0.8 ± 0.01	0.6 ± 0.04
C 22:5n-3	3.3 ± 0.08	2.4 ± 0.07	5.04 ± 0.03	5.9 ± 0.1	5.9 ± 0.1	5.1 ± 0.2	6.8 ± 0.1	5.9 ± 0.2	2.1 ± 0.02	1.5 ± 0.09
C 22:6n-3	8.2 ± 0.1	5.7 ± 0.2	1.06 ± 0.02	0.9 ± 0.02	0.4 ± 0.009	0.3 ± 0.02	1.3 ± 0.02	1.2 ± 0.04	0.3 ± 0.005	0.2 ± 0.003
∑ SFA	37.4 ± 0.6	39.6 ± 0.6	31.6 ± 0.2	32 ± 0.2	30.8 ± 0.1	32.4 ± 0.3	28.6 ± 0.2	31.1 ± 0.2	33.1 ± 0.2	36.8 ± 0.9
∑ MUFA	21.6 ± 0.9	23.4 ± 1.1	6.1 ± 0.1	12.1 ± 0.6	8.4 ± 0.2	10.1 ± 0.3	5.1 ± 0.08	6.9 ± 0.6	8.4 ± 0.04	9.5 ± 0.2
∑ PUFA**	40.9 ± 0.4	37.1 ± 0.5	62.3 ± 0.2	55.9 ± 0.7	60.8 ± 0.1	57.5 ± 0.5	66.3 ± 0.15	62.03 ± 0.7	58.4 ± 0.2	52.7 ± 0.8
∑ N6	28.3 ± 0.6	27.5 ± 0.3	50.4 ± 0.2	41.5 ± 0.5	39.7 ± 0.1	38.1 ± 0.3	46.9 ± 0.1	44.1 ± 0.4	54.3 ± 0.2	50.6 ± 0.7
∑ N3**	12.7 ± 0.2	9.6 ± 0.2	11.9 ± 0.08	14.3 ± 0.2	21.1 ± 0.07	19.5 ± 0.4	19.4 ± 0.1	17.9 ± 0.4	4.1 ± 0.04	3.1 ± 0.14
n-6/n-3° PUFA	2.9		2.9		1.95		2.5		16.3	
PUFA/SAT	0.94		1.74		1.8		1.99		1.4	

n-6/n3 PUFA ratio as well as PUFA/SAT were computed in the processed sample only as this was our focus.

** Significantly different between species (p all < 0.0001).

° Not significant (p > 0.05).

**Table 2:** FA composition of Total Lipids (TLs) extracted from pheasant, hare, red deer, roe deer and wild boar in a raw (R) and a processed (P) state; values are given in weight% with means ± SEM

	Pheasant		Hare		Red deer		Roe deer		Wild boar	
	R	P	R	P	R	P	R	P	R	P
N	5	5	6	6	6	5	6	6	5	6
C 14:0	1.05 ± 0.03	0.51 ± 0.06	1.72 ± 0.15	1.38 ± 0.2	7.40 ± 0.54	2.88 ± 0.21	1.24 ± 0.07	1.00 ± 0.21	1.72 ± 0.10	1.31 ± 0.04
C 15:0	0.59 ± 0.05	0.25 ± 0.03	0.52 ± 0.03	0.44 ± 0.02	1.02 ± 0.03	0.48 ± 0.03	0.98 ± 0.02	1.05 ± 0.13	0.35 ± 0.03	0.32 ± 0.03
C 16:0	26.19 ± 0.79	13.89 ± 1.30	9.19 ± 2.04	15.62 ± 1.40	6.09 ± 0.41	20.06 ± 1.35	17.28 ± 0.67	14.78 ± 1.53	4.13 ± 0.28	24.06 ± 0.26
C 17:0	0.60 ± 0.04	0.28 ± 0.03	0.71 ± 0.06	0.54 ± 0.02	0.80 ± 0.05	0.52 ± 0.04	1.34 ± 0.08	0.94 ± 0.13	0.40 ± 0.05	0.56 ± 0.02
C 18:0	12.63 ± 0.40	6.21 ± 0.58	5.19 ± 1.75	7.66 ± 0.32	3.35 ± 0.26	8.02 ± 0.62	20.70 ± 1.79	15.89 ± 2.74	2.59 ± 0.07	12.82 ± 0.13
C 16:1n-7	5.51 ± 0.42	2.04 ± 0.22	18.02 ± 3.36	1.65 ± 0.22	41.76 ± 2.16	4.83 ± 0.30	1.75 ± 0.14	0.98 ± 0.07	35.00 ± 1.63	3.19 ± 0.09
C 18:1n-9	26.01 ± 1.13	47.82 ± 4.66	9.86 ± 0.69	44.35 ± 2.40	11.90 ± 0.58	37.07 ± 1.89	11.30 ± 0.76	38.45 ± 2.23	14.08 ± 0.76	35.03 ± 1.16
C 18:2n-6	16.13 ± 0.80	19.09 ± 1.92	30.88 ± 0.20	16.14 ± 0.66	14.95 ± 0.82	16.43 ± 0.50	21.78 ± 1.49	15.14 ± 1.41	31.64 ± 2.02	17.79 ± 0.91
C 18:3n-3	1.90 ± 0.07	5.13 ± 0.49	4.29 ± 0.25	4.23 ± 0.63	4.49 ± 0.23	5.85 ± 0.26	4.47 ± 0.21	3.20 ± 0.15	1.40 ± 0.05	0.96 ± 0.03
C 20:4n-6	1.53 ± 0.22	2.11 ± 0.18	10.98 ± 2.01	4.14 ± 0.50	3.87 ± 0.32	1.71 ± 0.15	7.47 ± 2.03	4.39 ± 0.71	6.10 ± 0.32	2.62 ± 0.39
C 20:5n-3	5.89 ± 0.79	0.44 ± 0.09	4.53 ± 2.31	0.90 ± 0.11	1.88 ± 0.14	0.77 ± 0.07	7.12 ± 2.00	1.59 ± 0.26	0.72 ± 0.05	0.36 ± 0.03
C 22:5n-3	0.91 ± 0.18	0.81 ± 0.09	2.77 ± 0.47	2.15 ± 0.21	0.56 ± 0.04	1.06 ± 0.08	3.07 ± 0.76	1.88 ± 0.25	0.64 ± 0.06	0.67 ± 0.06
C 22:6n-3	1.07 ± 0.23	1.46 ± 0.15	1.39 ± 0.35	0.78 ± 0.04	2.14 ± 0.13	0.32 ± 0.03	1.50 ± 0.23	0.70 ± 0.09	1.39 ± 0.010	0.33 ± 0.03
∑ SFA	41.05 ± 0.87	21.13 ± 1.96	17.33 ± 3.79	25.64 ± 1.60	18.65 ± 0.25	31.96 ± 2.14	41.54 ± 2.62	33.67 ± 4.50	9.19 ± 0.37	39.06 ± 0.33
∑ MUFA	31.53 ± 1.55	49.86 ± 4.60	27.88 ± 3.63	46.00 ± 2.23	53.65 ± 1.87	41.90 ± 1.69	13.05 ± 0.64	39.42 ± 2.26	49.08 ± 2.35	38.22 ± 1.17
∑ PUFA**	27.42 ± 1.84	29.04 ± 2.88	54.84 ± 0.53	28.35 ± 1.30	27.90 ± 1.62	26.13 ± 0.77	45.42 ± 3.14	26.91 ± 2.83	41.87 ± 2.25	22.72 ± 1.39
∑ N6	17.66 ± 0.94	21.20 ± 2.09	41.86 ± 1.96	20.28 ± 1.11	18.82 ± 1.11	18.14 ± 0.52	29.25 ± 2.32	19.53 ± 2.11	37.73 ± 2.15	20.40 ± 1.26
∑ N3**	9.76 ± 0.91	7.84 ± 0.79	12.97 ± 1.70	8.08 ± 0.47	9.08 ± 0.52	7.99 ± 0.26	16.16 ± 1.66	7.37 ± 0.72	4.14 ± 0.22	2.32 ± 0.14
n-6/n-3° PUFA	2.70		2.50		2.27		2.65		8. 79	
PUFA/SAT	1.37		1.1		0.82		0.8		0.6	

** Significantly different between species (p all < 0.0001).

° Not significant (p > 0.05).

For diets to provide a valuable source of n-3 PUFA, they must meet two criteria: A high proportion of PUFA compared with SFA and MUFA and a high proportion of n-3 FA within PUFA. Indeed, most game meat samples in our model study had a high PUFA content even after processing (Tables 1, 2). Moreover, except for wild boar, the n-6/n-3 ratio in game meat was definitely below the ratio of 10–20:1 as can be seen from Figure 1a, b and Tables 1 and 2. The ratio of 10–20:1 is typical for the average Western diet so game meat is much closer to the ratio of 1:1 of our ancestors [5, 10]. This has also been shown earlier for several African game species [22]. Our new data also indicate that the cooking process, as typically used for game, does not significantly impair its desirable FA profile. Expectedly, processing slightly decreased PUFA content in both TL and PL, but these changes did not affect the n-6/n-3 balance at all (Table 1), mirroring results previously published concerning beef [24].

Our study, however, also points to the need to differentiate between game species (Tables 1, 2; Figure 1a, b) and to a higher variation than typically observed in domesticated species. One could question, however, if our data are representative for each species due to the relatively small amount of muscle tissue (6–10 g) analysed. However, we are confident in our results due to the following arguments. (1) (between species)—Fatty acid composition is a tightly regulated trait in mammals and PUFA content varies according to the animal’s taxonomy between 34.5 (cattle) and 70% (ibex) [25] and variation within a species is very low (c.f. SEM’s in Tables 1 and 2). By sampling six individuals per species we have attempted a comprehensive reflexion of the natural variability in game meat fatty acid composition. (2) (within species)—In the past, we have conducted several experiments on fatty acid composition within one species i.e., European hares (*Lepus europaeus*) where we studied three different muscles from 293 individuals with the differences in the content of PUFA and other FA classes being in the range of <5% [26]. Also, we studied different strains of laboratory mice and found that within species, or between different body locations the observed differences in PUFA contents or in PUFA subclasses such as n-3 or n-6 were very low [27, 28]. We thus consider muscle fatty acid composition shown here to be representative of each species.

## Conclusion

We conclude that game meat can provide a healthy dietary component, as processing does not substantially alter its favourable FA composition, with both the n-6/n-3 and P:S ratios falling within previously recommended levels (Tables 1, 2; Figure 1a, b). We however point out that distinguishing between species is requested as e.g. FA composition of wild boar is not as health promoting compared with, for instance, hare and roe deer. Previously published data on the PUFA/SFA ratio from pig, sheep and cattle muscle TL fatty acid composition amounted to 0.58, 0.15 and 0.11 respectively [23] and thus are somewhat lower than our data from Table 2 underlining our statement that game meat provides a high quality nutrient although we are aware that certain livestock, especially when kept on natural pastures may show favourable PUFA/SFA ratios as well [23]. Similarly, the n-6/n-3 PUFA ratio reportedly was 7.2, 1.3 and 2.1 for pig, sheep and cattle respectively according to Wood et al. [23], but was between 2.3 and 2.7 in all game species examined here except for wild boar (Table 2).

In this context it should be noted that, while game meat in Europe was previously often contaminated with lead or other chemicals, this is not the case anymore, except for wild boar in certain locations [29]. A further, added benefit of the consumption of game is that permissible harvesting of animals that live and forage freely in their natural habitats arguably meets a higher ethical standard of meat production in comparison to livestock husbandry.

## Methods

6–10 g of frozen skeletal muscle (Musculus longissimus *thoracis et lumborum* and *Musculus pectoralis* in pheasants) were obtained from Restaurant Mörwald “Zur Traube” in A-3484 Feuersbrunn am Wagram, Austria. All samples were handled and processed by the same chef throughout the study. From the original sample, we separated and analysed two 0.5 g muscle samples for both analyses of polar phospholipids (PL) and total lipids (TL), i.e., in a raw and processed state, from 6 individuals each in the following species: Pheasant (*Phasianus colchicus*), European hare (*Lepus europaeus*), Red deer (*Cervus elaphus*), Roe deer (*Capreolus capreolus*), and Wild boar (*Sus scrofa*). We only analysed processed meat samples that were ‘well done’ and contained no pink (non-fried) parts, with no added antioxidants and/or additives. Firstly, all muscle samples were homogenized and lipids were extracted by using chloroform and methanol (2:1 v/v), then either directly transesterified (for TL samples) or separated on silica gel thin layer chromatography plates (Kieselgel 60, F254, 0.5 mm, Merck, Darmstadt, Germany) before they were made visible under ultraviolet light with the PL fraction isolated. We are confident that the use of thin layer chromatography does not lead to a significant loss of PUFA by oxidation as both fatty acid content and profiles are congruent from solid phase extraction and thin layer chromatography. PL and TL extracts were transesterified by heating (100°C) for 30 min in acidic atmosphere (H_2_SO_4_ in Methanol), extracted into hexane and analysed by gas liquid chromatography (GLC) (Shimadzu GC 2010 with Autosampler and FID; Kyoto, Japan). FA methyl esters of a set of 13 FA were identified by comparing retention times with those of FA methyl standards (Sigma-Aldrich, St. Louis, USA) as outlined elsewhere [28] and by using GC Solution Analysis software (version 2.42.00, Shimadzu, Kyoto, Japan). Concentrating on the characterisation of the TL and PL lipid class, we used no internal standard for FA GC as the distribution of the lipid classes in our small sample was unknown.

Statistical analyses were conducted in R [30]. The FA contents and FA classes between species, as well as between raw and processed samples were compared, using repeated measurements Analysis of Variance in R [30]. To account for the fact that each individual was entered into the dataset twice (with the raw and processed meat sample), we applied linear mixed effects models with “individual” as a random factor.

## Abbreviations

DHA: docosahexaenoic acid
EPA: eicosapentaenoic acid
FA: fatty acid
GLC: gas liquid chromatography
MUFA: monounsaturated fatty acid
P: processed sample
PL: phsopholipid
PUFA: polyunsaturated fatty acid
R: raw sample
SFA: saturated fatty acid
TL: total lipid
